# Novel Unsaturated Polyester Nanocomposites via Hybrid 3D POSS-Modified Graphene Oxide Reinforcement: Electro-Technical Application Perspective

**DOI:** 10.3390/nano10020260

**Published:** 2020-02-03

**Authors:** Nidhin Divakaran, Manoj B. Kale, T. Senthil, Suhail Mubarak, Duraisami Dhamodharan, Lixin Wu, Jianlei Wang

**Affiliations:** 1 CAS Key Laboratory of Design and Assembly of Functional Nanostructures, and Fujian Key Laboratory of Nanomaterials, Fujian Institute of Research on the Structure of Matter, Chinese Academy of Sciences, Fuzhou, Fujian 350002, China; nidhin@fjirsm.ac.cn (N.D.); manojkale@fjirsm.ac.cn (M.B.K.); suhail@fjirsm.ac.cn (S.M.); duraisamidhamodharan@fjirsm.ac.cn (D.D.); 2 University of Chinese Academy of Sciences, Beijing 100049, China; 3 Advanced Research School for Technology and Product Simulation, Central Institute of Plastics Engineering and Technology, Chennai 600032, India; tsenthilsci@gmail.com

**Keywords:** graphene oxide, aminopropyl isobutyl POSS, in situ polymerization, unsaturated polyester, mechanical properties, electrical conductivity

## Abstract

The latest trends in technologies has shifted the focus to developing innovative methods for comprehensive property enhancement of the polymer composites with facile and undemanding experimental techniques. This work reports an elementary technique to fabricate high-performance unsaturated polyester-based nanocomposites. It focuses on the interactive effect of polyhedral oligomeric silsesquioxanes (POSS)-functionalized graphene oxide (GO) within the unsaturated polymermatrix. The hybrid framework of POSS-functionalized graphene oxide has been configured via peptide bonding between the aminopropyl isobutyl POSS and graphene oxide. The synergistic effect of POSS and graphene oxide paved the way for a mechanism to inculcate a hybrid framework within the unsaturated polyester (UP) via in situ polymerization to develop UP/GO-POSS nanocomposites. The surface-appended POSS within the graphene oxide boosted its dispersion in the UP matrix, furnishing an enhancement in tensile strength of the UP/GO-POSS composites by 61.9%, thermal decomposition temperature (10% mass loss) by 69.8 °C and electrical conductivity by 10^8^ S/m, in contrast to pure UP. In particular, the homogenous influence of the POSS-modified GO could be vindicated in the surging of the limiting oxygen index (%) in the as-prepared nanocomposites. The inclusive property amelioration vindicates the use of fabricated nanocomposites as high-performance nanomaterials in electrotechnical applications.

## 1. Introduction

The current revolution in polymer technologies reflects the role of thermosetting plastics in their advancement. The dexterous properties of the thermosetting plastics have empowered researchers to widen their possible application in a vast range of fields [1,2,3,4]. The enticing properties such as low viscosity (uncured state), customized modulus (according to application), high thermal stability, smooth process ability and their ability to use with fillers has provided impetus in expanding their commercial applications [5]. The recent global thermosetting market implies mushrooming demand of unsaturated polyester (UP)-based composites in industrial and commercial applications. However, the task of unsaturated polyester is subjected to various impediments such as invariably low thermal stability, poor electrical conductivity, inadequate chemical resistance etc. [6,7,8]. A plethora of research has been conducted to incorporate UP with fillers in order to augment their comprehensive properties [9,10,11]. Nevertheless, enhancement of some properties of UP imperils other vital properties, which could jeopardize the versatility of the polymer. This triggers an impulse to construct a hybrid material network within the polymer matrix, ultimately yielding the merits of the materials involved in polymer reinforcement. The materials could be nanofillers forming a hierarchical network within the polymer matrix, to facilitate interfacial bonding with the polymer [12]. Graphene-based hybrid materials are envisaged to be impeccable in the interfacial bonding with the polymer as well as assisting better dispersion in the matrix. Previous studies have employed the use of graphene, in addition to other fillers, as a hybrid material for the polymer matrix reinforcement [13,14]. Graphene oxide (GO) (oxide form of graphene with functional groups) has the property of being a versatile nanofiller owing to its remarkable reciprocity with the polymer matrix [15,16]. GO sheets are chemically modified with functionalization agents or modifiers to make it compatible with the polymer matrix and enhance the interfacial inter-linkage with the polymer matrix [17].

Polyhedral oligomeric silsesquioxane (POSS) is a three-dimensional nanostructured material with excellent inclusive properties, possessing cage-like molecular structure [18,19]. POSS is delineated using the formula (RSiO_1.5_)_n_, where R = organic (alkyl, aryl) or hydrogen group. POSS, having a diameter 1–3 nm, consists of the Si–O linkage as a backbone in the cubical cage with the Si atom on each of its vertex connecting the O atom. The functional group attached to the POSS molecules tailor its properties and plays a very important role in the POSS-polymer interaction. The hybrid POSS material has a significant role in the property enhancement of the polymer such as thermal stability, mechanical strength, flame retardancy etc., as shown in the works reported earlier [20,21,22,23,24,25]. This led to a logical inference of amalgamating the stellar properties of GO by modifying it with POSS. The hybrid POSS-GO material could consolidate in the dispersion within the polymer matrix, thereby ameliorating its properties. Zhang et al., implemented this concept by examining the degree of mechanical reinforcement of POSS-GO within the cyanate ester composites, obtaining 56% enhancement in flexural strength with 0.6 wt% POSS-GO addition [26]. Zhang et al. developed a hybrid network of POSS-GO by using 1,6-hexanediamine as bridge and surveyed their synergistic effect in epoxy matrix, achieving 29.29% improvement in impact strength and a 63.16% surge in thermal conductivity of epoxy matrix [27]. Hu et al. observed a 2.5-fold increase in tensile strength of waterborne polyurethane composites with 0.20 wt% POSS-GO addition thereby vindicating the remarkable interaction of POSS-GO within the polymer matrix [28]. Raimondo et al. utilized the hybrid structure of POSS-graphene nanosheets within the epoxy resin as aircraft lighting strike protection [29]. However, no works have been reported untilnow on the combined effect of POSS-GO within the unsaturated polyester matrix. A lot of research has been carried out to investigate the interactive effect of nanofillers with UP matrix. Lin et al. adopted an in situ method to develop halloysite nanotubes/silica nanohybrid and examined their interaction within UP matrix [30]. Jiang et al. attained the interfacial reinforcement of UP nanocomposites by incorporation of hybrid POSS-carbon fibers to procure mechanical properties optimization [31]. Wu et al. examined the interfacial interaction of carbon nanotubes with UP carbon fiber composites and surveyed the interlaminar studies [32]. Liu et al. observed the influence of a low addition of graphene into the bio-based UP resins, evaluating its thermo-mechanical properties [33]. The interaction of other nanofillers with the UP matrix proved to be a catalyst in pursuit of exploring the impact of POSS-GO in the UP matrix.

Our work follows the novel route of fabricating the unsaturated polyester nanocomposites by incorporating POSS-modified GO within the polymer matrix by in situ polymerization. Limited prior research works had focused on the addition of the functionalized GO within the unsaturated polyester matrix [34,35], but none had adopted the solvent-free in situ polymerization technique. Aminopropylisobutyl POSS has been used as an organic modifier to functionalize with the GO. Different wt% of the nanofillers are supplemented into the UP-polymer matrix during in situ polymerization. The subsequent nanocomposites are subjected to thermal, mechanical and electrical property analysis to inspect the influence of the hybrid POSS-GO in the UP. The limiting oxygen index values of the nanocomposites are also monitored to gauge the alteration in flame-retardancy phenomena. The comprehensive property evaluation has inferred culminative outputs stating the overall improvement of UP polymer with diminutive addition of nanofillers. The prerequisite of achieving high-performance materials with excellent comprehensive properties for electrotechnical application has been encompassed here. The inclusive property amelioration of UP has propelled the scope of its application in the electrotechnical field, mainly due to its electrical conductivity being thoroughly enhanced, along with its flame retardancy, thermal stability and mechanical strength. The thermal conductivity enhancement within the POSS-GO-reinforced UP has paved the path for less heat dissipation and appropriate thermal management in electrotechnical devices [36].

## 2. Experimental

### 2.1. Materials

Aminopropylisobutyl polyhedral oligomeric silsesquioxanes (POSS-NH_2_) was purchased from Passkey Technology Co. Ltd. (Changsha, Hunan, China). Dicyclohexylcarbodiimide (DCC) was obtained from Alladin Industrial Co. Ltd. (Shanghai, China). Graphite was purchased from Alladin Chemicals Ltd. (98%) (Shanghai, China). Anhydrous Tetrahydrofuran (THF) was supplied by Sigma-Aldrich (Shanghai, China). NaNO_3,_ KMnO_4_, H_2_O_2_, and ethanol were purchased from Alladin Industrial Corporation (Shanghai, China). Phthalic anhydride (PA) and maleic anhydride (MA) were obtained from Alladin Industrial Corporation (Shanghai, China). Propylene glycol (PG) and ethylene glycol (EG) were procured from General Reagent (Shanghai, China). Diethylene glycol and styrene were obtained from Xilong Corporation (Guangdong, China). Cobalt iso-octanoate was procured from Shengfei Fine Chemical plant (Shanghai, China) and methyl ethyl ketone peroxide (MEKP) was obtained from AkzoNobel Corporation (Shanghai, China), both are used as catalyst and initiator respectively. All chemicals were used as received without any purification.

### 2.2. Synthesis of Graphene Oxide(GO)

Graphene oxide was synthesized from graphite powder using modified Hummer’s method [37]. Initially 3 g graphite, 3.6 g sodium nitrate and 140 mL concentrated sulphuric acid were supplemented into the three-necked round bottom flask kept in an ice bath and mixed together using a mechanical stirrer at 300 rpm for 30 min; 18 g of KMnO_4_ was added slowly and reacted for 6 h at 35 °C to transform the graphite intercalation compound into graphite oxide. Subsequently, 150 mL of deionized water was added slowly over 30 min and the temperature was retained between 0 and 5 °C for 10 h. Then, 10 wt% H_2_O_2_ solution was added to cease the reaction. The solution was washed several times using distilled water until the pH became 7 and after centrifugation the solids were obtained.

### 2.3. Synthesis of Polyhedral Oligomeric Silsesquioxane(POSS)-Functionalized GO (POSS-GO)

The synthesis route to functionalize the GO with POSS-NH_2_ is depicted as follows: 50 mg of GO, 50 mg of DCC and 2.0 g of POSS-NH_2_ were dissolved in 50 mL of anhydrous THF. The above mixture was charged in a two necked flask endowed with a nitrogen and a condenser. DCC acts a catalyst in this process for the peptide bond synthesis between COOH group attached to GO and NH_2_-functionalized POSS. The mixture solution was subsequently ultrasonicated for 1 h. The solution obtained was magnetically stirred and refluxed for 48 h under a nitrogen atmosphere. The stirring was ceased every 8 h and the solution was stowed in the ultrasound bath for a proper dispersion for 10 min. The obtained product was subjected to vacuum filtration and later washed with THF to remove the untreated POSS-NH_2_ with the final product having a yield of 79.5% (100 mg).

### 2.4. Synthesis of Pure Unsaturated Polyester (UP)

The development of pure UP resin was formulated using melt polycondensation reaction. The synthesis route of UP preparation has been adopted from the literature with some modifications [38]. Initially, 16 g PG, 20.5 g EG, 32.5 g DEG, 72.5 g PA, 27.5 g MA were mixed and charged in a three necked flask with stirring equipment and gradually heated till the temperature reaches 120 °C. Subsequently, 0.1 wt% antioxidant (triphenylphosphite) was added into the mixture and the temperature was raised to 160 °C after the solid PA and MA were completely melted. The actual polycondensation reaction commences at 160 °C and is processed between 160 °C and 210 °C followed by checking of the acidic value. On the reduction of the acidic value of the UP/mixture to 70 mg KOH/g, the reaction was processed under vacuum at 210 °C till the acidic value was lower than 26 mg KOH/g. Later the temperature was reduced to 190 °C and 0.01 wt% (to that of total resin). Hydroquinone was added into the mixture. The reactive diluent styrene (31 wt%) was added to the resin as the temperature approached 120 °C. The UP pre-polymer was assorted with cobalt iso-octanoate and MEKP for free radical polymerization to undergo curing process. It was followed by pouring of the mixture on a Teflon mold. The curing process was carried out for 3 h in room temperature and post-cured at 3 h at 80 °C in a vacuum oven.

### 2.5. Synthesis of UP/POSS-GO Nanocomposites via In Situ Polymerization

**Figure 1:**
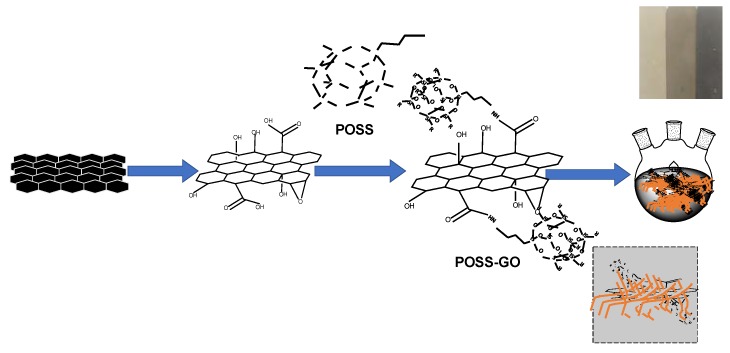
Schematic route to covalently functionalize graphene oxide (GO) using polyhedral oligomeric silsesquioxanes (POSS) and synthesize unsaturated polyester (UP)/POSS-GO nanocomposites: (1) modified Hummers method, (2) POSS functionalization using peptide bonding, (3) in situ incorporation along with UP preparation after sonication with glycols, (4) Preparation of nanocomposites after curing(color variance of nanocomposite from pure UP to 0.05 UP/POSS-GO to 0.10 UP/POSS-GO indicating superior dispersion with increase in wt%).

Figure 1 exhibits the synthesis scheme of the preparation of UP/POSS-GO nanocomposites. The various concentrations of GO-POSS (0.05%, 0.08%, 0.1%, and 0.3%) were added to the glycols used in the preparation of UP. The glycol mixture was subjected to ultra-sonication for 1 h. Later, the dispersed glycol/POSS-GO mixture along with other chemicals used in the UP-preparation procedure were added into a three-necked rounded beaker endowed with a magnetic stirrer and the whole UP preparation procedure was followed.

### 2.6. Characterization

Fourier transform infrared spectra (FTIR) analysis was carried out using a Perkin Elmer Spectrum One Spectrometer (Waltham, MA, USA) with KBr from 500 to 4000 cm^−1^ with a resolution of 4 cm^−1^. The X-ray Diffraction (XRD) analysis was performed using Rigaku Miniflex600 (Tokyo, Japan) processed with Cu-Kα radiation (λ = 1.5406 A^°^) under the testing condition of 2θ from 5°–50°. X-rayphotoelectron spectroscopy (XPS) was conducted using a Thermo Scientific ESCALAB 250Xi^+^ Spectrometer (Waltham, MA, USA). Raman spectroscopy was done using Renishaw, Invia (West Dundee, IL, USA) with the wavelength of the laser being 514 nm. Scanning electron microscopy (SEM) was performed using HITACHI, SU8010/EDX (Tokyo, Japan) with Energy disperse X-Ray spectrometer.

Transmission electron microscopy (TEM) was conducted using JEM (JE-2010 OL, Tokyo, Japan) with the accelerating voltage of 200 kV. The samples were made by depositing a drop of solution on a copper grid with thin film formvar. Thermogravimetric analyses (TGA) were done using TA Instruments STA449C (Milford, MA, USA) in the range of 25–800 °C under a nitrogen atmosphere with the heating rate of 10 °C/min. Thermal conductivity was investigated using the TC 3000 thermal conductivity tester (Xiatech Instrument Factory, Xi’an, China) using the transient hot wire method and the sample size being 3 cm × 1 cm. The tensile properties were surveyed using a universal testing machine (AGS-X PLUS, Shimadzu, Kyoto, Japan) according to ASTM D638 and ASTM D790 standards respectively. The average values of a set of five specimens of each nanocomposite was considered. Dynamic mechanical analysis (DMA) was performed on a TA DMA Q800 apparatus from TA Instruments (Milford, MA, USA). A single cantilever mode was used for testing. DMA tests were performed from 30 °C to 180 °C with a heating rate of 3 °C/min at 1 Hz. Electrical conductivity measurement of the samples was carried using a four-point or Kelvin probe method (Nanometrics, USA). The specimens were cut to the size of 10 × 10 mm^2^, coated on both sides with electrodeposited silver paste (100 nm thick). The silver paste enabled the sample to develop Ohmic contacts on both sides assisting the computation of the electrical conductivity in the Kelvin probe. Limiting oxygen index (LOI) values were examined using the Stanton Redcraft flame meter (Shanghai, China), along with oxygen. The sample analysis was executed according to the ASTM D2863/77 standards. The vertical burning tests were conducted in the flame meter, with the dimensions of the sample size being (10 ± 0.002) × (10 ± 0.002) × (0.3 ± 0.002) cm^3^. Each sample was buckled vertically in the center of the column and burned for a period of 3 min.

## 3. Results and Discussion

### 3.1. Characterization of GO and POSS-GO

**Figure 2:**
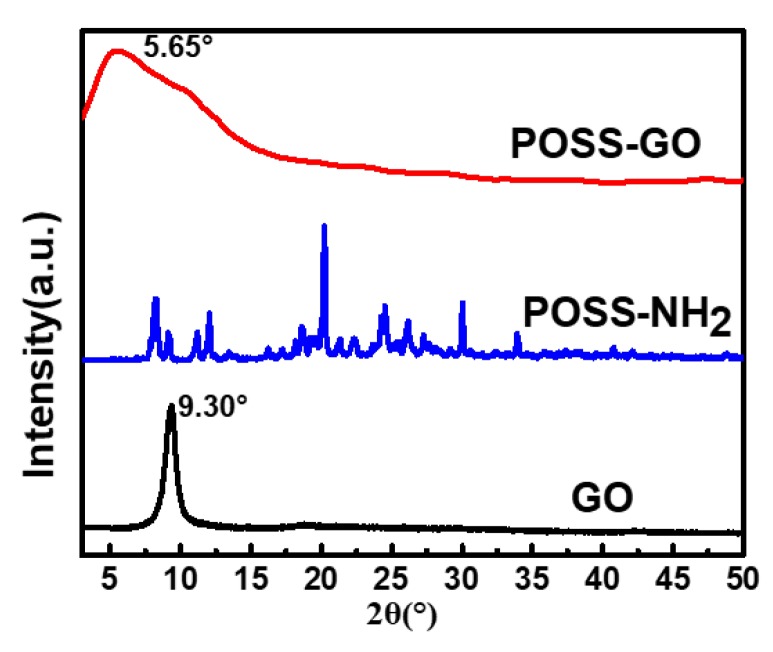
X-ray Diffraction (XRD) outputs for GO, POSS-NH_2_ and GO-POSS.

The successful functionalization of graphene oxide using POSS is vital in the interfacial reinforcement within the polymer matrix. Figure 2 displays the XRD patterns which substantiates the successful functionalization of GO using POSS. The POSS comprise of the amine groups attached on its surface. The XRD diffraction peak of GO is obtained at 2θ = 9.30°, corresponding to the inter layer spacing of 0.9250 nm. The XRD analysis of POSS-NH_2_ displays numerous diffraction peaks at different angles, depicting its crystalline structure. The XRD diffraction peak of POSS-GO furnishes the successful exfoliation of the GO using POSS, with the characterization peak at 2θ = 5.65°. The interlayer distance within the GO increased to 1.56 nm, which reiterates the exfoliation of GO due to the covalent bonding between POSS-NH_2_ onto the surface of GO [39,40]. The presence of POSS on the surface of GO shields the aggregation of the GO nanoparticles in the polymer matrix, thereby assisting in immaculate interfacial interaction with the polymer.

**Figure 3:**
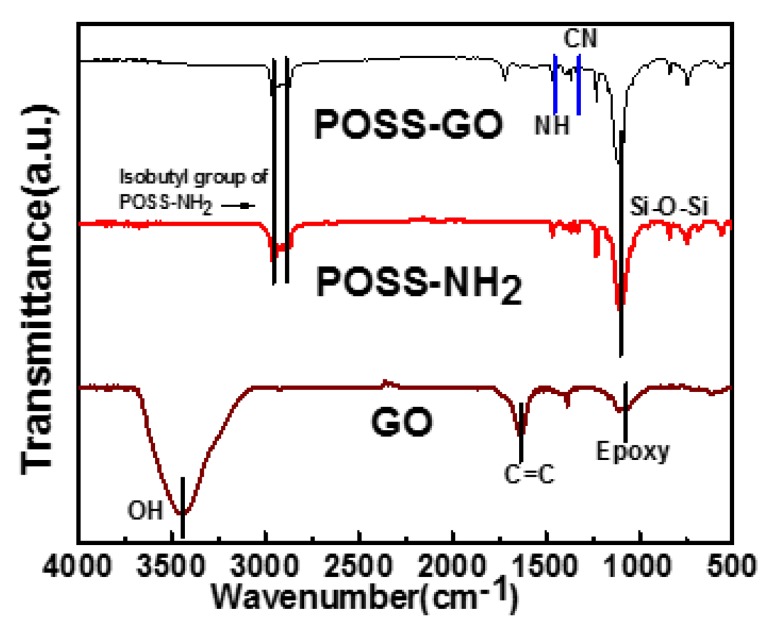
Fourier transform infrared spectra (FTIR) inference for GO, POSS-NH_2_ and GO-POSS.

FTIR analysis confirmed the successful covalent modification of graphene oxide using POSS. Figure 3 showcases the FTIR spectra for GO, POSS-NH_2_ and POSS-GO. The FTIR peaks of GO exhibited predictable outputs, with peaks at 1063 cm^−1^ (epoxy group), 1618 cm^−1^ (C=C bond) and the presence of hydroxyl (OH) groups at 3400 cm^−1^. The FTIR peaks of POSS-GO hybrid displayed perceptible changes due to the covalent bonding between POSS moiety and GO. There exists a peak at 1102 cm^−1^ due to the Si–O–Si stretching band, imbibed from the POSS-NH_2_ also vindicated in its FTIR peak [41,42]. There is a substantial absorption peak at 2870 cm^−1^ in POSS-GO FTIR spectra, obtained from the isobutyl groups of POSS-NH_2_. Some peaks are also visible at 1402 cm^−1^ and 1234 cm^−1^ in POSS-GO due to presence of N–H in plane and C–N bond stretching, respectively [28]. The existence of these groups in POSS-GO validates the covalent bonding of POSS-NH_2_ with the GO. 

**Figure 4:**
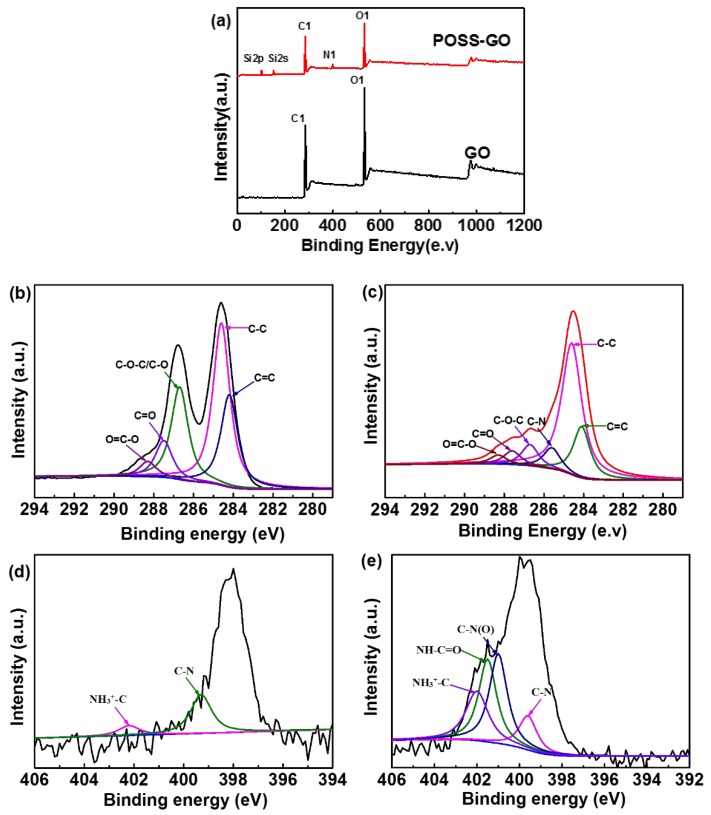
(**a**) X-ray photoelectron spectroscopy (XPS) survey peak of GO and POSS-GO, high resolution C1 spectra of (**b**) GO and (**c**) POSS-GO. High resolution N1 spectra of (**d**) POSS-NH_2_ and (**e**) POSS-GO.

The formation of the hybrid-network of POSS-GO depends on the appropriate surface functionalization of GO using POSS-NH_2_. Figure 4a shows the XPS survey peak spectra of GO and POSS-GO. The survey peak of GO corroborates the presence of high-intensity O1 spectra (532 eV) and C1 spectra (284.6 eV). The survey peak of POSS-GO showcases additional peaks Si 2s (152.3 eV), Si 2p (102.5 eV) and N spectra (399.9 eV) [41,43]. The new auxiliary peaks perceived in XPS survey analysis of the POSS-GO depicts their presence in the functional groups appended to the POSS. These peaks are procured due to the functionalization of GO using POSS-NH_2_, describing successful covalent modification. Detailed XPS analysis using the deconvolution technique was carried out to further confirm the covalent modification of GO using POSS-NH_2_ with the high resolution C1s spectra of GO and POSS-GO, as specified in Figure 4b,c respectively. The high resolution C1s spectra of GO displays several oxygenated peaks such as C=O peak at 287.5 eV, O−C=O peak at 288.3 eV, C=C peak at 284.1 and C–O–C peak at 286.6 eV. The presence of oxygen moiety in the GO generates the scope of further functionalization to constitute functionalized GO and this contrives the impulse to covalently modify GO using POSS [44,45]. This could be substantiated from high resolution C1s spectra of POSS-GO. The spectra convey the diminishing of the peak intensity of C−O−C at 286.6 eV and C=O at 287.5 eV. This could be attributed to the deoxygenation of the GO due to the presence of nucleophilic substitution between POSS-NH_2_ and GO. There is an additional peak arising at 285.6 eV due to the presence of the C−N bond in POSS-GO [46]. The O−C=O peak at 288.8 eV also faded due to the covalent bonding with the amino isobutyl groups of the POSS [47]. Figure 4d,e represents the high resolution N1s spectra of POSS-NH_2_ and POSS-GO, respectively. The N1 spectra of POSS−NH_2_displays peak arising at 399.6 eV due to the presence of N−C(pyrrolic) bond of the aminopropyl group and at 402 eV owing to the presence of NH_3_^+^-C bond [48,49]. The peak at 399.6 eV reduces in N1 spectra of POSS-GO and becomes N–C (pyridinic) bond [50]. There is a slight increase in the NH_3_^+^–C bond peak (402 eV) on interaction of POSS with GO, while there is additional peak at 401 eV due to the N–C(O) (graphitic) bond [43]. An apparent peak at 401.5 eV is visible in the N1s spectra of POSS-GO specifying the covalent bond existing between POSS and GO due to attachment of nitrogen to carbonyl group (amide bond (NH−C=O)) [51]. The inference of successful covalent modification of GO using POSS could be vindicated from the XPS analysis.

**Figure 5:**
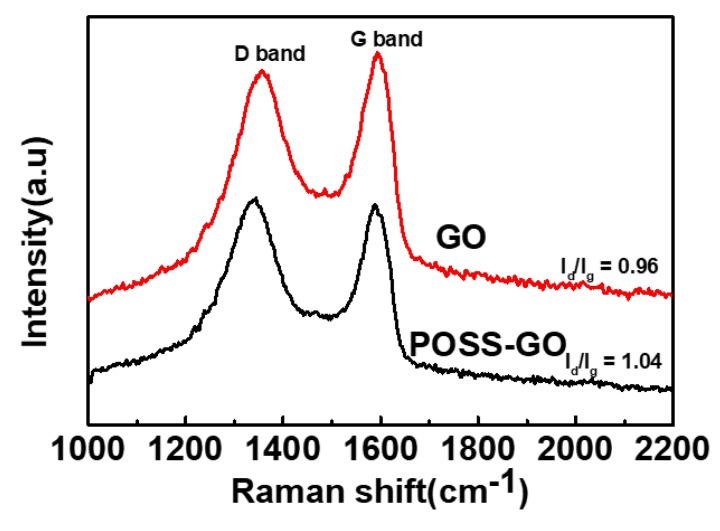
Raman spectra analysis of GO and POSS-GO, with I_d_/I_g_ ratio values representing the extent of functionalization of GO using POSS.

The Raman spectroscopy technique furnished cogent analysis of the covalent substitution existing between GO and POSS-NH_2_. Figure 5 exhibits the Raman spectroscopy outputs of GO and POSS-GO. Both GO and POSS-GO displayed peaks around at D band (1340 cm^−1^) and G band (1585 cm^−1^). The existence of disoriented amorphous carbon atoms in the carboniferous materials such as GO generates the D band, while the G band emerges due to the sp^2^ hybridized carbon atoms in the hexagonal structure within GO [52]. The I_d_/I_g_ ratio derived from the Raman spectra analysis could determine the degree of functionalization of GO using POSS [53]. Figure 5 conveys a minor surge in I_d_/I_g_ ratio in POSS-GO Raman spectra which could be ascribed to the covalent functionalization of GO using POSS. The slight improvement in I_d_/I_g_ ratio corroborate the evolution of surface edge defects on GO due to its covalent modification using POSS and the reduced size of the sp^2^ realm within GO [54].

**Figure 6:**
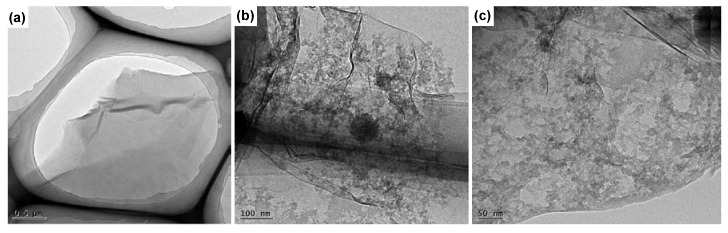
Transmission electron microscopy (TEM) images of (**a**) GO and (**b**) POSS-GO with (**c**) higher magnification image of POSS-GO.

The surface morphological analysis of GO and POSS-GOusing TEM has been conducted to investigate the impact of the functionalization of GO using POSS. The successful formation of GO sheets with profoundly oxygenated functional groups could be vindicated from the smooth surface displayed in Figure 6a. The laminar and wavy structure observed in the TEM image of GO is due to epoxy, hydroxyl and carboxyl groups attached onto the GO surface. The POSS functionalized GO presented a comparatively rougher surface with the GO being covalently modified using POSS. Figure 6b,c exhibit innumerable dark spots onto the surface of GO which could be attributed to the successful modification of GO using POSS, paving the way for the possible reinforcement in the UP matrix [44].

### 3.2. Thermal Properties of UP/POSS-GO Nanocomposites

**Figure 7:**
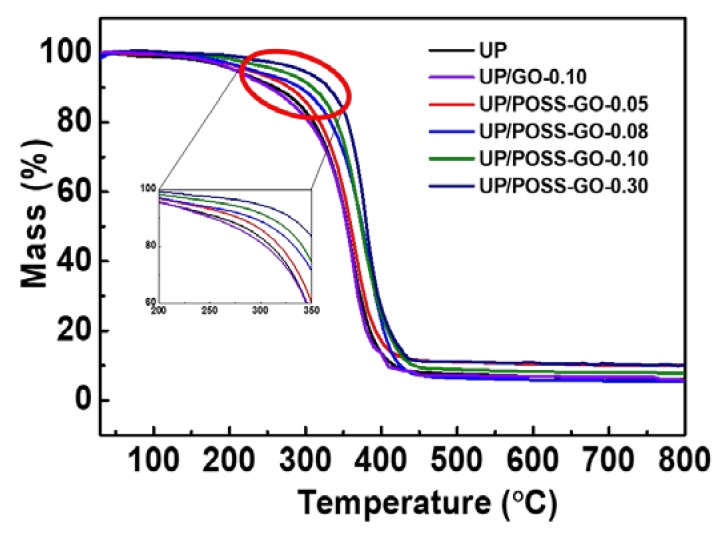
Thermogravimetric analysis (TGA) of UP, UP/GO and UP/POSS-GO nanocomposites with the inset depicting the enlarged view of thermal degradation being undertaken.

A thorough overview regarding the thermal degradation properties of the nanocomposites, with pure UP composite as reference could be retrieved from TGA analysis and the results have been summarized in Table 1. Figure 7 deduces tiny weight loss for UP and its nanocomposites at the temperature range of 100 °C and 150 °C, attributed to the evaporation of absorbed water. The subsequent weight loss at the temperature range around 275 °C crop up due to the pyrolysis of oxygen functional groups, CO_2_ and water vapor. This weight loss appears to be less in UP/POSS-GO composites as compared to pure UP and UP/GO composites. Table 1 implies the vivid inference of the amplification of T_10_ and T_50_ values for UP/POSS-GO composites.There seems to be less improvement of thermal degradation temperature values for UP/GO composite, clearly substantiating the inability of pristine GO to interact within the UP-polymer matrix [55]. The T_10_ value of UP/POSS-GO composites has been remarkably enhanced with the value of UP/POSS-GO-0.30 increased by 69.8° C to that of pure UP. The POSS-functionalized GO is validated as being a mass transfer barrier against the volatile components within the UP matrix, impending the thermal decomposition of UP [56]. The ensuing phenomenon witnesses the single step transition thermal decomposition roughly at 350 °C for UP and their nanocomposites. This occurs due to the breaking of bonds within the polymer. However, this phenomenon is a bit delayed in the UP/POSS-GO nanocomposites as the interaction of POSS-GO within the matrix has created hurdle for the polymer chains mobility, deferring the breaking of bonding in the polymer. Table 1 concludes the amelioration of T_50_ values of UP/POSS-GO composites, thereby affirming the logic of a synergistic effect of POSS and GO within the UP matrix. There appears to be enhanced residual weight (%) for UP/POSS-GO composites at 800 °C, inferring the role of meticulous POSS-GO dispersion within the UP matrix.

**Figure 8:**
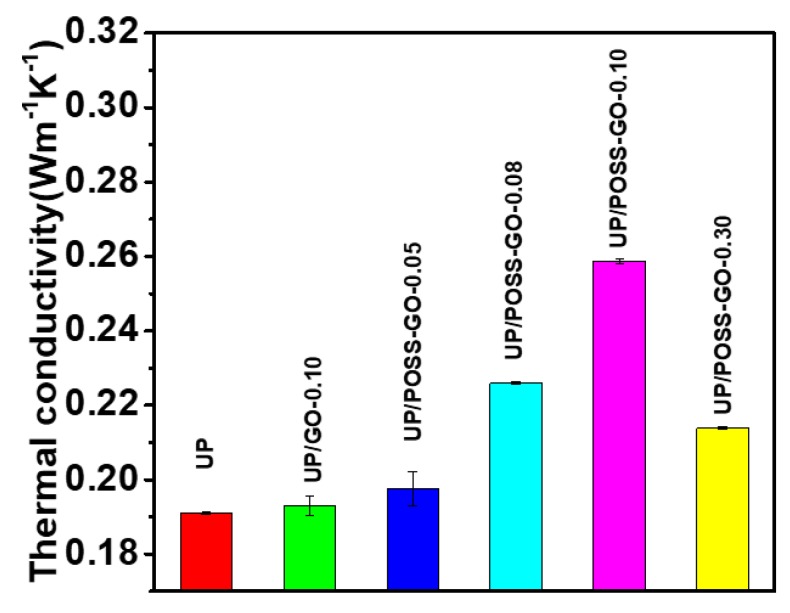
Thermal conductivity analysis of the pure UP, UP/GO and UP/POSS-GO nanocomposites.

The thermal conductivity values, as specified in Figure 8 and Table 1, has increased with the addition of different wt% of POSS-GO within the UP matrix. The thermal conductivity of UP/GO composite do not display any enhancement because of irregular interaction of pristine GO with the UP-polymer matrix. The combined impact of POSS and GO has resulted in the augmentation of the thermal conductivity of UP, surmounting the low intrinsic thermal conductivity of POSS [57]. The UP-polymer matrix, devoid of free electrons, solely depends on phonons for the transfer of thermal energy. These phonons traverse across the amorphous and defect-rich surfaces of the polymer, thereby diminishing the comprehensive thermal conductivity. There prevails an urge to incorporate UP with crystalline nanofillers such as POSS, with an aim to sustain the phonon transport mechanism through the crystalline lattice and enhancethe free volume in the polymer matrix [58]. The low intrinsic thermal conductivity of POSS has been overcome with the presence of GO and their collegial influence has resulted in an immaculate interaction with the polymer matrix and the formation of a bridge for the transport of phonons. However, there is a reduction in thermal conductivity at 0.30 wt% POSS-GO, interpreting the agglomeration of the nanofillers with UP-polymer matrix. This critically affects the phonon transport in the polymer. The steady improvement in thermal conductivity values of the as-prepared nanocomposites at diminutive addition of POSS-GO conceives a blooming path for the utility of these nanocomposites in thermal management devices and electrotechnical applications.

### 3.3. Morphological Properties of UP/POSS-GO Nanocomposites

**Figure 9:**
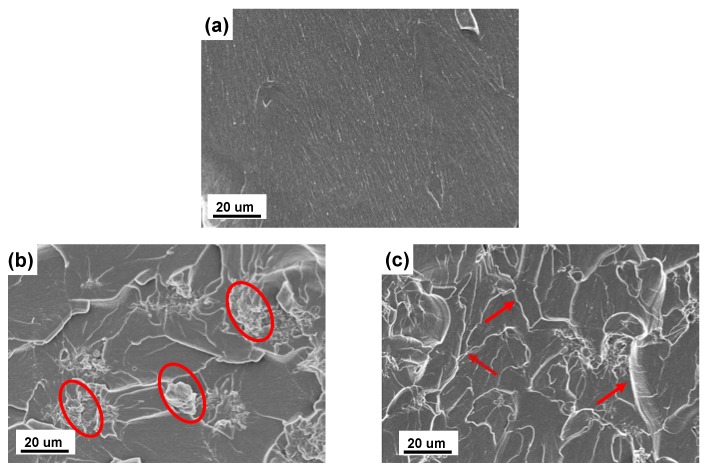
Scanning electron microscopy (SEM) images of (**a**) UP, (**b**) UP/ POSS-GO-0.05 and (**c**) UP/POSS-GO-0.10 nanocomposites. The red circles in b represents the protruded-out GO particles, owing to feeble incorporation in UP matrix. The red arrow marks in c depicts the directions of crack propagation due to presence of POSS-GO in the UP matrix.

SEM analysis of the fractured surfaces of the UP and UP/POSS-GO nanocomposites was conducted to examine the scrupulous dispersion of POSS-GO within the UP matrix. The SEM image of the fractured surface of UPexhibits smooth surface and there appears to be very tiny cracks which are too small to be visible, while SEM images of UP/POSS-GO nanocomposites possess cracks on the surface with the presence of nanofillers within the polymer matrix. Figure 9b presents a rough surface structure morphology with few protruding POSS-GO particles, as marked with a red circle. The particles extending onto the surface of UP prevail due to the presence of POSS-GO aggregates in the polymer, creating improper dispersion of nanofiller within the polymer at low concentration of 0.05 wt% [59]. The roughening of the polymer surface occurs with the addition of POSS-GO and the crack-propagation during fracturing of the surface is constrained. This notion is coherent with the mechanical property enhancement of the nanocomposites with the initiation of crack pinning and crack deflection mechanism. Figure 9c exhibited a better dispersion of POSS-GO into the UP matrix with the increase in wt% (0.10 POSS-GO). The red arrow marks in Figure 9c denoted the crack propagation with miniscule bulged-out POSS-GO particles. It provides clear evidence of astrong tendency of POSS-GO to interact with the polymer matrix and influencethe comprehensive properties of nanocomposites.

### 3.4. Mechanical Properties of UP/POSS-GO Nanocomposites

**Figure 10:**
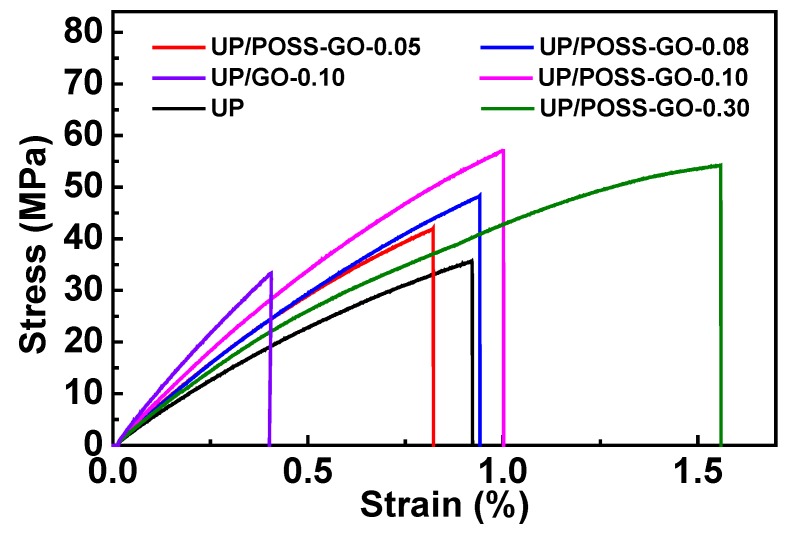
Stress-strain inference results for UP, UP/GO and UP/POSS-GO nanocomposites.

The mechanical properties’ analysis of the nanocomposites, in terms of tensile strength measurement, was executed with different wt% of POSS-GO within UP matrix, as shown in Figure 10. The tensile strength of UP ameliorated considerably with the incorporation of POSS-GO. The logic behind the tensile strength enhancement of nanocomposites relies on the thorough exfoliation of GO using POSS, enabling their synergistic effect on the local stress concentration for potent load transfer from polymer matrix to nanofillers. The auxiliary rationale relies on the covalent modification of GO using POSS and their immaculate interaction with the UP matrix. The UP/GO nanocomposite displays no improvement in the tensile strength and elongation at break (%), vindicating the poor interaction of pristine GO with the UP matrix. The incorporation of POSS-GO into the UP matrix via in situ polymerization imparts superior reinforcement efficiency than pristine GO. The tensile strength values tend to decrease with the addition of 0.30 POSS-GO into UP matrix which could be ascribed to the agglomeration of POSS-GO above the critical concentration. It influences the inclusive strength of the polymer with sporadic local stress concentration induced by the clustering of the POSS-GO particles. The tensile strength of UP/0.10 POSS-GO was boosted by 61.90% with respect to pure UP, verifying the conclusion from the SEM images outputs (Figure 9c). The increasing trend in elongation at break (%) values for UP with wt% ranging from 0.08 POSS-GO to 0.30 POSS-GO apparently conceives the role of the nanofiller in augmenting the maximum stress endurance of the polymer. The collegial effect of POSS and GO has ceased the polymer chain mobility which inflates the elongation at break (%) values with the increase in concentration of POSS-GO.

**Figure 11:**
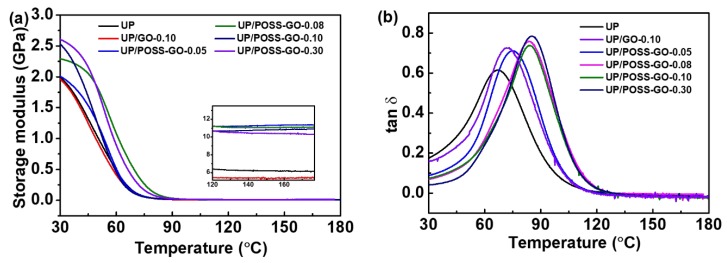
Dynamic mechanical analysis (DMA) outputs for (**a**) storage modulus of UP, UP/GO and UP/POSS-GO and (**b**) tan *δ* representation for UP, UP/GO and UP/POSS-GO. The inset in (**a**) demonstrates storage modulus at high temperature (120 °C up to 180 °C) for the nanocomposites.

DMA analysis was performed on UP and its nanocomposites to evaluate their thermo mechanical properties and the viscoelastic behavior in presence of POSS-GO. According to the Figure 11a, the storage modulus declines with the temperature surge due to softening of UP polymer chains. The storage modulus values at 30 °C, as viewed in Table 2, enhance with the increase in wt% of POSS-GO, furnishing a strong reinforcement effect prevalent in UP with the addition of nanofillers. There appears to be a mild influence of pristine GO addition in polymer matrix, providing a conjecture about their feeble interaction with the polymer as the storage modulus and glass transition temperature of UP/GO nanocomposite tend to possess slight enhancement. The highly crystalline POSS functionalized to GO has boosted the storage modulus of UP polymer as high as 2600 MPa, which is 32.4% enhancement with regard to pure UP. POSS-GO acts as a reinforcing agent which enables competent stress load transfer, thereby composing stiffness within polymer and enhancing their storage modulus [60]. This has culminated in the enhanced molecular mobility within the UP matrix. However, the higher wt% of 0.30 POSS-GO exhibited dwindling storage modulus at high temperature attributed to the presence of adequate amount of nanofillers restricting polymer chain movement. Figure 11b displays the tan delta peak deviating to the right side with the addition of nanofillers. The enhancement in glass temperature demonstrates strong interaction between POSS-GO andthe UP matrix. The presence of crystalline POSS in GO has averted the blocking effect generated by GO, emanating soaring molecular mobility in polymer chains. This led to an increase in T_g_, affirming a strong crosslinking of nanofillers with UP. The proper exfoliation of GO due to POSS has also propelled the viscoelastic properties improvement of the nanocomposites.

### 3.5. Electrical Properties of UP/POSS-GO Nanocomposites

**Figure 12:**
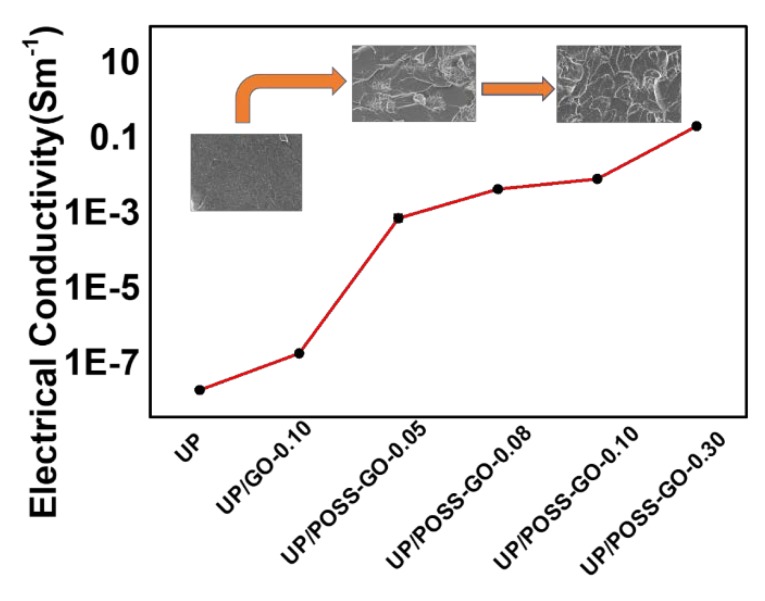
Electrical conductivity of UP, UP/GO and UP/POSS-GO nanocomposites. The inset displays the scanning electron microscopy (SEM) images (see Figure 9) which co-relates with conductivity enhancement, where the increase in wt% of POSS-GO has resulted in proper dispersion simultaneously boosting electrical conductivity.

The survey of electrical properties of nanocomposites is vital in analyzing their electrotechnical application features. Thermal management of the polymer matrix must be in tandem with their electrically conductivity aspects. The profound interaction of the nanofiller with UP matrix is critical for a material to inculcate electrical conductivity. The pristine GO raises the electrical conductivity, as mentioned in Figure 12 and Table 3, due to incomplete formation of conducting link by poor interaction with UP. The superior aspect ratio of POSS-GO plays a significant role in propelling up the electrical conductivity by creating a conducting network within UP matrix [61]. The addition of POSS-GO enhances the electrical conductivity values by 10^8^ S m^−1^ as compared to pure UP. The detriment of POSS being an insulator has been abated with the presence of GO, as the POSS-GO amalgamation established a network for the flow of electrons, contributing to electrical conductivity [62]. The increase in fillers loading in the UP has elevated its electrical conductivity with the mechanism of hopping, tunneling and conduction occurring in the polymer matrix. The pertinent POSS-GO fills up the insulating gap in polymer and bolster the route for electron mobility. The agglomeration of the nanofillers at higher concentration of POSS-GO do not impinge on the overall electrical conductivity as their uneven distribution has generated unmitigated contacts, creating a sufficient path for the electrons to flow. The surge in electrical conductivity of UP at a minimal addition of POSS-GO, vindicates the aggrandizement of polymer application in electrotechnical field to a greater extent.

### 3.6. Limiting Oxygen Index of UP/POSS-GO Nanocomposites

**Figure 13:**
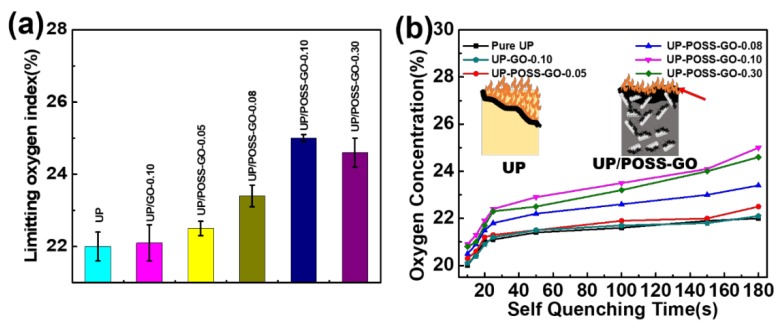
(**a**)LOI values of UP, UP/GO and UP/POSS-GO nanocomposites. (**b**) Oxygen concentration (%) values with different self-quenching time for the nanocomposites. The inset in (**b**) displays the synergistic effect of POSS-GO in retarding the flame. The red arrow represents the carbonaceous char produced due to the presence of POSS-GO, resisting the flame.

The LOI technique is one of the important factors to determine the flame-retardancy potential of polymer nanocomposites. It regulates the ability of the polymer to decelerate the flame on burning. The samples are positioned in a candle-like configuration within the flame meter. The addition of pristine GO to UP, as shown in Figure 13a and Table 2, showcases a meagre effect on LOI values confirming the inability of pristine GO to interact with the UP matrix. The ability of UP to extinguish appears to be faster than that of UP/POSS-GO nanocomposites. The self-quenching time of UP, as in Figure 13b, exists at lower oxygen concentration. The LOI values favored to increase with the addition of POSS-GO. The presence of POSS moiety attached to GO mobilized towards an impeccable interaction with the UP matrix. The three dimensional network of POSS validates in being amiable to the flame retardant mechanism of polymer by increasing its viscosity on heat exposure [63]. The nanohybrid POSS-GO obstructs the release of volatile products of the polymer on exposure to flame. Hence they impact the overall flame retardancy of the nanocomposites through the “Labyrinth effect” mechanism [64]. The increment in LOI values of nanocomposites also converges with the elevation of oxygen concentration at different self-quenching time. However, at a certain concentration of 0.30 wt% POSS-GO, the LOI value tends to decrease due to agglomeration of nanofillers within the polymer. This leads to intermittent configuration of fillers against the raging flame and their efficiency to retard flame degrades. The superior thermal conductivity of GO and excellent compatibility with the UP matrix due to POSS, plays an important role in improving the flame-retardant mechanism [65]. The comprehensive property enrichment of UP incorporated with POSS-GO shores up the feasible approach of a polymer to restrict the flame and boost the LOI values for fulfilling the criteria for electrotechnical applications [66].

## 4. Conclusions

A facile technique to develop UP/POSS-GO nanocomposites has been adopted. The hybrid network of POSS-GO was configured via peptide bonding. The combined effect of POSS-GO in property enhancement of a UP polymer matrix was investigated by incorporating POSS-GO into UP through in situ polymerization. The thermal stability for UP at 0.30% POSS-GO addition was enhanced by 69.8 °C, as compared to pure UP. The hybrid POSS-GO acted as a barrier against thermal degradation of the polymer matrix. The tensile strength also demonstrated improvement by 61.9% for 0.10 wt% POSS with reference to pure UP due to the synergistic impact of POSS-GO in the stress load transfer towards polymer matrix. Moreover, the nanocomposites cropped up with elevated thermal conductivity in addition to its improved electrical conductivity (by 10^8^ S m^−1^ as compared to UP). Thermal management devices demand the prerequisite of padding the polymeric material retained with flame retardancy. The LOI values (important aspects of flame retardancy) of UP/POSS-GO nanocomposites were ameliorated with an increase in the amount of nanofiller (POSS-GO). The 3D POSS exhibited immaculate combination with GO to resist the flame, thereby enhancing the LOI. The combined influence of POSS as well as GO propelled the inclusive property enhancement of the UP matrix. These nanocomposites could be combined with fibers to be developed as fiber-reinforced plastic for commercial applications in the near future. There arises scope for a plethora of research to be undertaken in enhancing the versatility of UP for electrotechnical applications.

## Figures and Tables

**Table 1:** Thermogravimetric analyses (TGA) and thermal conductivity measurement of UP/GO and UP/POSS-GO nanocomposites.

Sample Name	*T*_10_ * (°C)	*T*_50_ * (°C)	Residual (%)	Thermal Conductivity (W·m^−1^ K^−1^)
UP	259.7	354.6	5.68	0.1911 ± 0.00038
UP/GO-0.10	255.4	352.2	5.89	0.1930 ± 0.0024
UP/POSS-GO-0.05	273.4	357.8	10.10	0.1976 ± 0.0045
UP/POSS-GO-0.08	293.3	376.4	5.99	0.2260 ± 0.0004
UP/POSS-GO-0.10	309.1	379.6	7.88	0.2587 ± 0.0005
UP/POSS-GO-0.30	329.5	380.6	10	0.2139 ± 0.00042

* *T*_10_ and *T*_50_ represents the temperature during which 10% and 50% mass loss happens.

**Table 2:** Mechanical and thermo-mechanical analysis of UP/GO and UP/POSS-GO nanocomposites.

Sample Name	Tensile Strength (MPa)	Elongation at Break (%)	Storage Modulus at 30 °C (MPa)	*T*_g_ * (°C)
UP	35.2 ± 0.5	0.92 ± 0.008	1963	69.5
UP/GO-0.10	32.3 ± 1.5	0.4 ± 0.100	1913	71.2
UP/POSS-GO-0.05	41.9 ± 3.2	0.82 ± 0.009	1982	74.9
UP/POSS-GO-0.08	48.3 ± 2	0.94 ± 0.012	2287	83.6
UP/POSS-GO-0.10	57 ± 1.4	1 ± 0.033	2516	84.7
UP/POSS-GO-0.30	54.1 ± 1.5	1.56 ± 0.044	2600	85.2

* *T*_g_ denotes the glass transition temperature.

**Table 3:** Electrical conductivity and limiting oxygen index (LOI) values of UP/GO and UP/POSS-GO nanocomposites.

Sample Name	Electrical Conductivity (S m^−1^)	LOI (%)
UP	2.12 × 10^−8^	22 ± 0.4
UP/GO-0.10	2 × 10^−7^	22.1 ± 0.5
UP/POSS-GO-0.05	7.87 × 10^−4^	22.5 ± 0.2
UP/POSS-GO-0.08	0.0047	23.4 ± 0.3
UP/POSS-GO-0.10	0.00878	25 ± 0.1
UP/POSS-GO-0.30	0.223	24.6 ± 0.4
